# ﻿Diving into Diversity: *Hasleaberepwari* (Bacillariophyceae, Naviculaceae), a new species of marine diatom from New Caledonia

**DOI:** 10.3897/phytokeys.255.144697

**Published:** 2025-04-25

**Authors:** Fiddy Semba Prasetiya, Thierry Jauffrais, Mustapha Mohamed-Benkada, Nolwenn Callac, Dominique Ansquer, Elif Yılmaz, Claude Lemieux, Monique Turmel, Jean-Luc Mouget, Danang Ambar Prabowo, Debora Christin Purbani, Diah Radini Noerdjito, Varian Fahmi, Sulastri Arsad, Romain Gastineau

**Affiliations:** 1 Research Center for Biosystematics and Evolution, Research Organization for Life Sciences and Environment, National Research and Innovation Agency (BRIN), Jalan Raya Bogor Km 46, Cibinong, West Java 16911, Indonesia Research Center for Biosystematics and Evolution, Research Organization for Life Sciences and Environment, National Research and Innovation Agency (BRIN) Cibinong Indonesia; 2 Ifremer, IRD, Univ Nouvelle-Calédonie, Univ La Réunion, CNRS, UMR 9220 ENTROPIE, BP 32078, 98800, Nouméa, New Caledonia Ifremer, IRD, Univ Nouvelle-Calédonie, Univ La Réunion, CNRS, UMR 9220 ENTROPIE Nouméa New Caledonia (Fr); 3 Laboratory of Aquaculture and Bioremediation, Faculty of Nature and Life Sciences, University Oran1-Ahmed Ben Bella P.B. 1524 El M’Naouer 31000 Oran, Algeria University Oran1 Oran Algeria; 4 Faculty of Nature and Life Sciences, University of Sciences and Technology- Mohamed Boudiaf, El Mnaouar BP 1505, Bir El Djir 31000, Oran, Algeria University of Sciences and Technology- Mohamed Boudiaf Oran Algeria; 5 Institute of Marine and Environmental Sciences, University of Szczecin, Mickiewicza 16a, 70-383 Szczecin, Poland University of Szczecin Szczecin Poland; 6 Institut de Biologie Intégrative et des Systèmes, Université Laval, Québec, Québec City, Canada Université Laval Québec Canada; 7 Laboratoire Biologie des Organismes, Stress, Santé, Environnement (BiOSSE), Le Mans Université, Avenue Olivier Messiaen, 72085 Le Mans, France Le Mans Université Le Mans France

**Keywords:** Coral Sea, Naviculales, organellar genomes, tropical diatoms

## Abstract

The current article introduces and describes *Hasleaberepwari***sp. nov.**, a new species of diatom discovered in the vicinity of Boulouparis, New Caledonia. Under light microscopy, *H.berepwari***sp. nov.** strongly resembles *Hasleapseudostrearia*, but preliminary molecular barcoding conducted using partial 18S and *rbcL* genes suggested that it was a distinct species. This was confirmed first by scanning electron microscopy which showed the differences in stria densities between both species. A short-reads genome-skimming protocol applied on *H.berepwari***sp. nov.** led us to obtain its complete mitochondrial and plastid genomes. The mitogenome is 36,572 bp in length and as already observed among other species of *Haslea* spp., the *nad6* and *nad2* genes are fused within a single open-reading frame. The plastome is 131,897 bp length, and unlike the mitogenome, it is not colinear with those of *H.pseudostrearia*. The results derived from the sequencing of the plastome allowed to perform a 123-gene multigene maximum likelihood phylogeny that associates *H.berepwari***sp. nov.** to *H.pseudostrearia* with maximum support at the nodes but also strictly distinguishes them, suggesting a greater genetic distance between these species than what has been previously observed between other marennine-producing species.

## ﻿Introduction

The genus *Haslea* comprises a group of morphologically diverse pennate diatoms, currently including 36 taxonomically accepted species (Guiry and Guiry 2011). The holotype species of the genus is *Hasleaostrearia* (Gaillon) Simonsen 1974, also known as the ‘blue diatom’ due to its capacity to produce blue pigment commonly known as marennine (Simonsen 1974). Marennine is responsible for the green coloration observed in the gills of oysters along the Atlantic coast of France (Gaillon 1820; Gastineau et al. 2014). Green oysters, which are distinguished by their specific flavor attributed to modifications in fatty acids and their emerald hue, are less common and command a higher price in the French oyster industry (Prasetiya et al. 2017a). While the chemical structure of Marennine remains unknown, it has been shown to display several biological properties (Pouvreau et al. 2007; Pouvreau et al. 2008; Gastineau et al. 2012b; Prasetiya et al. 2016; Prasetiya et al. 2017b; Prasetiya et al. 2019a; Falaise et al. 2019a, 2019b; Permatasari et al. 2019; Prasetiya et al. 2020a; 2020b; Prasetiya et al. 2021a, 2021b; Seveno et al. 2024). Latest research and development efforts have focused on scaling up the production of the diatom and its pigment (Gargouch et al. 2022; Nghiem Xuan et al. 2021; Adjout et al. 2022; Prasetiya et al. 2022).

In recent years, several new species of *Haslea* have been described worldwide, some of which also produce blue pigments, while others exhibit shapes unusual for this genus (Poulin et al. 2004; Gastineau et al. 2012a, 2016, 2021b; Talgatti et al. 2014; Sterrenburg et al. 2015; Li et al. 2017; Prasetiya et al. 2019b; Lobban et al. 2020). Among the species that do not produce a blue pigment, *Hasleapseudostrearia* Massé, Rincé & E.J. Cox 2001 is of particular relevance to the present article. This species was first described by Massé et al. (2001) based on material from the Kingsbridge estuary in southern UK and received its species name due to its morphological similarities with *H.ostrearia*. Following this initial description, strains of non-blue *Haslea* were assigned to this species after being reported in various and distant parts of the world, including South Africa (GenBank: OK729589 and OK729583) and the Yellow Sea (An et al. 2017) (GenBank: KY320350 and KY320289).

The present study aims to describe a new species of pennate non-blue diatom from the genus *Haslea* originating from New Caledonia. New Caledonia, a territorial French collectivity, comprises several archipelagos and isolated islands, some of which are remnants of the Zealandia submerged continent. New Caledonia is situated approximately 1,500 km east of Australia, in the Southwestern Pacific Ocean and its largest island is named Grande Terre (‘great land’). On the west coast of Grande Terre, in Boulouparis, the French national institute for ocean science and technology (Ifremer) has operated a station for experimental aquaculture for approximately 50 years (Galinie 1989) (currently co-operated with the Adecal Technopole) with decades of expertise in shrimps and other crustaceans’ cultivation (e.g. for recent developments Lemonnier et al. 2021; Giraud et al. 2021, 2022; Colette et al. 2022, 2023; Nguyen et al. 2022; Callac et al. 2022, 2023, 2024), including experiments on the co-cultivation of shrimps with holothurians (Purcell et al. 2006; Bell et al. 2007). In 2020, a strain of non-blue *Haslea* has been isolated from one of the earthen ponds used in these studies. Under light microscopy (LM), the strain looked very similar to *H.pseudostrearia*, and if it were not for the contradictory results of molecular barcoding, it could have been assigned to this species.

In this article, we describe the new species *Hasleaberepwari* sp. nov. This description is based on LM and scanning electron microscopy (SEM) observations combined with two multigene phylogenies derived from the sequencing of the mitochondrial and plastid genome of this new species. The interest of New Caledonia as a hotspot for the discovery of new diatom species, as illustrated by this article, will be also discussed.

## ﻿Material and methods

### ﻿Sampling site, isolation and culture condition

**Figure 1.**
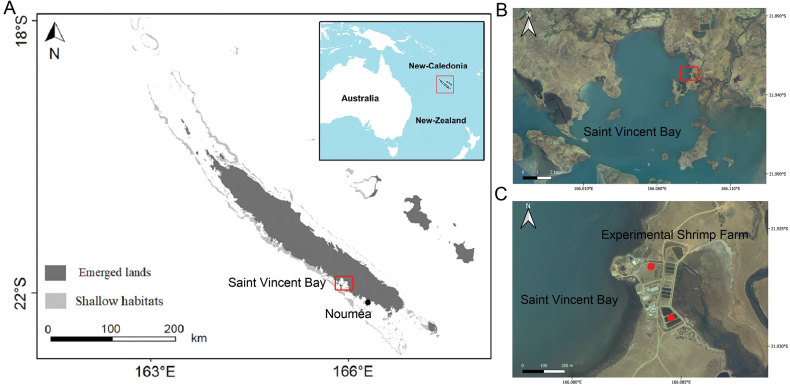
Sampling location of *Hasleaberepwari* in the experimental aquaculture station of Saint Vincent (21°55'36.9"S, 166°05'00.9"E), the West Coast of Grande Terre, New Caledonia, during summer 2020. Sampling point is indicated with the red dots sign. Map produced with QGIS software version 3.43.3 (QGIS.org 2023).

Samplings were done in August 2020 in shrimp earthen ponds use for the co-breeding of *Penaeusstylirostris* Stimpson, 1871 and *Holothuriascabra* Jaeger, 1833 at the experimental aquaculture station of Saint Vincent (Boulouparis), located on the west coast of Grande Terre (coordinates: 21°55'36.9"S, 166°05'00.9"E, sampling authorization 15569-2019/4-ISP-DENV) (Fig. 1). Monoclonal cultures were obtained by single cell isolations performed with micropipettes under an inverted microscope (Zeiss, Primovert). Subsequently, it was carefully rinsed in several drops of site-filtered seawater (0.2 µm). The isolated strain was then transferred to cell culture multidishes filled with filtered (0.2 µm), autoclaved, and ES1/3-enriched seawater (Lebeau et al. 1999). The isolates were then cultured in a thermo-controlled incubator at a temperature of 24 °C, with an illumination of 50 µmol photons m^-2^ s^-1^. Finally, the isolates were transferred to 150 mL Erlenmeyer flasks containing 50 mL of ES1/3 medium and maintained under similar conditions in our culture collection. The strain was registered in the collections of Ifremer as P05.

### ﻿Microscopic observations

Live LM images were obtained using a Leica microscope (DM750, Leica Microsystems) equipped with ICC50 camera. The morphometry analysis was conducted using the Image J software (Schneider et al. 2012). All LM pictures were taken within two weeks after isolation, isolation and come as close as possible to wild or natural material.

For cleaned LM and SEM observations, cells were subsequently rinsed in two consecutive baths of milliQ water and left to oxidize overnight in H_2_O_2_. The following day, a drop of the sample was deposited on a glass cover slide, air-dried, and placed in a furnace (2 hours at 450 °C) for complete removal of organic matter. Pictures were taken with the same Leica DM750 microscope mentioned above.

For scanning electron microscopy (SEM) observations, cleaned samples were coated with an 8 nm layer of platinum (LEICA, EM ACE 600) and imaged using an environmental SEM equipped with a secondary electron detector (JSM-IT300 LV, 20 kV, 15 mm working distance, JEOL) at the University of New Caledonia.

### ﻿DNA extraction and preliminary molecular barcoding

A culture of *H.berepwari* sp. nov. was maintained in exponential growth phase by frequent dilutions with fresh medium. A volume of 10 mL was harvested by centrifugation at 4500g and 5 °C during 10 min (Firlabo, SW9R, Meyzieu, France). Pellets of cells were then stored at -20 °C until analysis. The PowerSoil® DNA Isolation Kit was then used to extract the DNA and lyse the frustules. The DNA amplification and sequencing protocols were adapted from Gastineau et al. (2016). In brief, PCR reactions were conducted in a final volume of 20 μl using hot-start Taq polymerase (1X) (Qiagen) with its buffer: 10X PCR buffer (Qiagen), 200 μM of a dNTPs mix (Promega). Two sets of primers were employed: one targeting the 18S rRNA gene (18SHASLEAF: 5’_CTGCCCTATCAGCTTTGGATGG_3’, 18SHASLEAR: 5’_CCATTCAATCGGTAGGTGCG_3’) and the *rbcl* gene (RBCLF: 5’_GTCTCAATCTGTATCAGAACGG_3’, RBCLR: 5’_CGGTTAGCTGTTGGTGTTTCAGCG_3’) at a final concentration of 0.2 µM for each primer. All amplifications were performed in a Veriti^TM^ thermocycler (Applied Biosystems, USA) for 35 cycles, as follows: 1 min denaturation at 95 °C, 1 min annealing at 60 °C for the 18S rRNA gene and 59 °C for the *rbcL* gene, 1 min 30 s elongation at 72 °C, followed by a final elongation step at 72 °C for 7 min. The size of all amplicons was verified using agarose gel electrophoresis. The PCR products that matched the expected size (around 1300 bp for the 18S rRNA gene and 1400 bp for the *rbcL* gene) were sent to GenoScreen (Lille, France) for Sanger sequencing. The raw sequence chromatograms were checked using Geneious Prime software and the reverse and forward sequences were combined. The consensus sequences were pairwise aligned using MEGA 11 (Tamura et al. 2021) and compared with sequences of *Haslea* spp. obtained from GenBank, which suggested that the species was different from these references. Consensus sequences are available as supplementary files as explained below.

### ﻿Next generation sequencing, assembly and annotation

The pool of DNA that remained from the PCR and Sanger protocol described above was sent to the Beijing Genomics Institute (BGI, Shenzhen, China) to be sequenced on a DNBSEQ platform for a total of ca. 100M clean 150 bp paired-end reads. Reads were assembled using SPAdes 4 (Bankevich et al. 2012) with a k-mer of 125. The different subunits of the plastome were joined using Consed (Gordon and Green 2013). Genes were identified and annotated as explained in Gastineau et al. (2021a). The maps of the mitochondrial and plastid genomes were drawn on the OGDRAW online portal (Lohse et al. 2013).

### Multigene phylogeny

Two phylogenies were performed. The first one was based on the available plastid genomes. Protein-coding genes were extracted from the plastomes of *H.berepwari* sp. nov. and 17 other species of *Naviculaceae* downloaded from GenBank plus *Eunotianaegelii* Migula 1905 to be used as an outgroup. Genes that were not shared by all the selected taxa or were likely pseudogenes were removed from the dataset, leading to a total of 123 conserved genes, which were all independently aligned by MAFFT 7 (Katoh and Standley 2013) with the -auto option, then trimmed with trimAl (Capella-Gutiérrez et al. 2009) and the -automated1 option before being concatenated by Phyutility 2.7.1 (Smith and Dunn 2008). The best model of evolution was verified on the concatenated alignment using ModelTest-NG (Darriba et al. 2020), which returned the GTR+I+G as best model with the three modes (BIC, AIC and AICc). The maximum likelihood phylogeny was performed using IQ-TREE 2.2.0 (Minh et al. 2020) with 1000 ultrafast bootstrap replicates. The second multigene phylogeny was performed by appending recently published datasets (Yılmaz et al. 2024a, 2024b) with sequences from *H.berepwari* and other *Haslea* spp. The dataset contains three genes, namely *psbC*, *rbcL* and *18S*. The phylogeny was conducted using the same software as above, but the best model of evolution was evaluated on each gene alignment separately prior to concatenation, and was chosen based on the BIC mode. The model chosen were GTR+I+G4 (*psbC*), TIM3+I+G4 (*rbcL*) and TrN+I+G4 (*18S*). The dataset was partitioned based on these models, with 1000 ultrafast boostrap replicates. The dataset, partition file and complete tree are available as described in the data availability statement.

## ﻿Results

### ﻿Taxonomy

#### ﻿ Haslea berepwari

Taxon classificationPlantaeNaviculalesNaviculaceae

Mouget, Gastineau & Jauffrais sp. nov.

A588B0D1-9EEF-5FC2-A22F-85751A5FCA3B

##### Type material.

**Figure 2.**
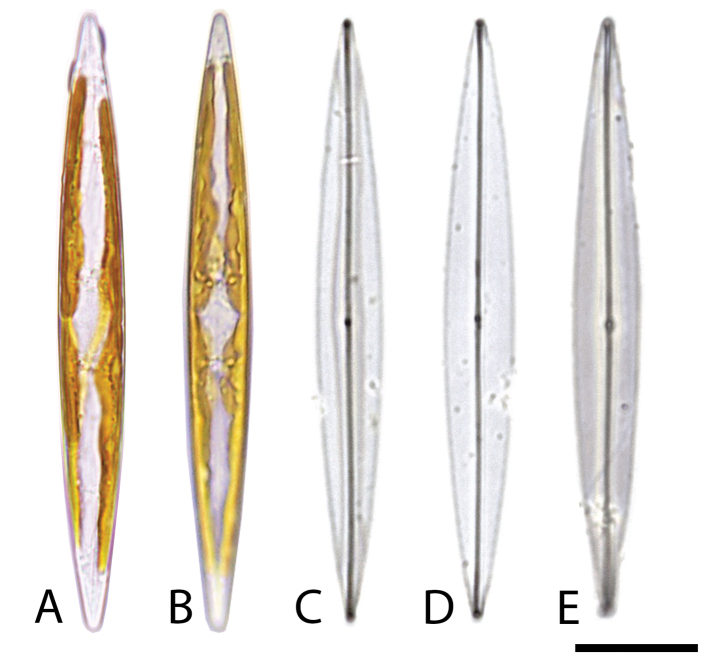
*Hasleaberepwari* sp. nov. *in vivo* pictures with two parietal chloroplasts and apices without the presence blue pigment in light microscopy (**A, B**) and LM image of a cleaned valve (**C–E**), scale bar 20µm.

***Holotype***: The strain P05 was acid cleaned and mounted on a glass slide and is deposited in the herbarium “Paris Cryptogamie” (PC) at The French National Museum of Natural History under accession number PC0643624 (LM slide) and PC0643625 (SEM slide). The cell representative of the type is presented in Fig. 2.

***Isotypes***: SEM and LM slides with acid cleaned valves of strain P05 are kept at the Ifremer culture collection in New Caledonia under the accession number P05.

##### Type locality.

Boulouparis, New Caledonia. *Hasleaberepwari* was isolated from shrimp earthen ponds (coordinates: 21°55'36.9"S, 166°05'00.9"E, Fig. 1) by Thierry Jauffrais in August 2020 in Boulouparis during a co-culture experiment of *Penaeusstylirostris* and *Holothuriascabra*.

##### Etymology.

The species designation is derived from the term “Boulouparis”, which is the one of the main cities on the west coast of New Caledonia. The name “Berepwari” is the translation of Boulouparis in xârâcùù, one of the main Melanesian languages spoken in New Caledonia.

##### Description.

*LM* Living cells solitary, motile and lanceolate, equipped with two parietal, narrow band-like chloroplasts appressed to the girdle of the cell (Fig. 2). Valves narrow and lanceolate with acute apices. The maximum and minimum length of the monoclonal culture of *H.berepwari* was 101.0 μm and 95.4 μm, respectively (average 98.0 ± 1.5 μm, n = 30), while the maximum and minimum width was 15.0 μm and 9.7 μm (average 12.2 ± 1.1 μm, n = 30). On clean frustules, raphe straight with non-distinct central endings. Cell wall exceedingly delicate, with longitudinal and transapical striations not discernible under LM. In general, LM provides minimal visibility into the specifics of the valve characteristics and is not sufficient to distinguish between this species and *H.pseudostrearia*.

**Figure 3.**
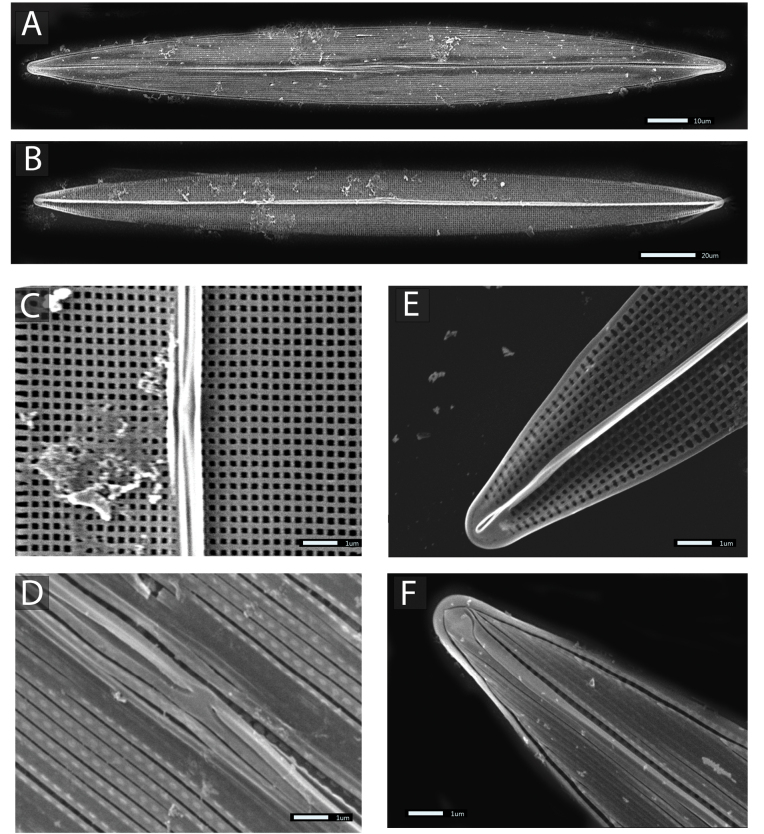
SEM micrographs of *Hasleaberepwari* sp. nov. strain P05. **A** Whole valve in external view **B** whole valve in internal view **C** internal view of the center of the valve demonstrating accessory ribs and the presence of a thin bar on the left side of the raphe **D** external view of the valve centre showing approximate raphe endings and continuous longitudinal fissures **E** internal view of apex with the helictoglossa **F** external view of apex showing the curved distal raphe fissure and part of the parallel and longitudinal slits adjoining the peripheral one. Scale bars: 10 μm (**A**); 20 μm (**B**); 1 μm (**C–F**).

*SEM* In external valve view, the exterior is covered with long, continuous, and apical-oriented siliceous stripes (top layer), proximal raphe endings straight and slightly widened, slightly deflected dorsally, apical raphe endings ventrally hook shaped (Fig. 3A, D). The interior is composed of a grate-like layer of small areolae, separated by short bars arranged crosswise. Transverse bars of this layer are almost equal in the transapical and longitudinal bars. The areolae are occluded externally by hymens and remnants of this membrane are visible in Fig. 3D. The central area lacks a lateral extension (Fig. 3A, D). Internally, the raphe is slightly elevated and straight, with well-developed helictoglossae at the poles (Fig. 3E). Internal openings of the raphe fissures directed towards one side of the raphe sternum, except at the center and near the tips. Thin bar near the central ending of the raphe on one side of the valve only (Fig. 3C). A supplementary ridge runs alongside the raphe sternum across most of the valve. Internally, square-shaped areolae organized in orderly rows (Fig. 3B, C, E). Externally, the valve seems covered with longitudinal bands, separated by slits running parallel to the raphe and converging into a single peripheral slit near the tips (Fig. 3A, F). The striation displays a transapical pattern of 37–38 striae per 10 µm intersected by a longitudinal pattern of 36 striae per 10 µm.

##### Differential diagnosis.

A comparative analysis of morphological features between *H.berepwari*, *Hasleanusantara* (Mouget, Gastineau and Syakti) and *H.pseudostrearia* is detailed in Table 1. *Hasleaberepwari* sp. nov. shares strong similarities with *H.pseudostrearia* but is distinguished from it by the density of striae, both transapical and longitudinal.

**Table 1.** Comparison between *H.berepwari*, the similar species *H.pseudostrearia* and the tropical species.

Features	*H.nusantara*	*H.pseudostrearia*	*H.pseudostrearia*	*H.berepwari*
Length (μm)	73.9 ± 1.7	55.5 ± 0.2	37–43	98.0 ± 1.5
Width (μm)	6.8 ± 0.1	8.8 ± 0.1	6–7	12.2 ± 1.1
Transapical striae in 10 μm	36.0 ± 1.0	38.6 ± 0.2	34–36	36
Longitudinal striae in 10 μm	52.0 ± 2.0	42.8 ± 0.2	42	37–38
Pseudostauros	Not present	Not present	Not present	Not present
Axial costa	Present	Present	Present	Present
Central bar	Present	Present	Present	Present
Central raphe endings	Straight	Straight	Straight	Straight
Polar raphe endings	Straight	Deflected	Deflected	Deflected
Presence of blue pigment	Yes	No	No	No
References	Prasetiya et al. (2019b)	Prasetiya et al. (2019b)	Massé et al. (2001)	in this study

##### Genomics and phylogeny.

***The nuclear rRNA gene cluster***: For reasons unknown, we failed to assemble the complete cluster of nuclear rRNA, even after adjusting the k-mer parameter for assembly. However, we successfully retrieved the complete 18S gene and submitted it to GenBank (PP725422). This sequence completely validated the results obtained previously from Sanger sequencing. The sequence was aligned using Clustal Omega (Sievers et al. 2011) with references ascribed to *H.pseudostrearia* (AY485524 and KY320350) and identity was respectively 95.12% and 95.30%, while these two references were 99.81% identical with each other.

**Figure 4.**
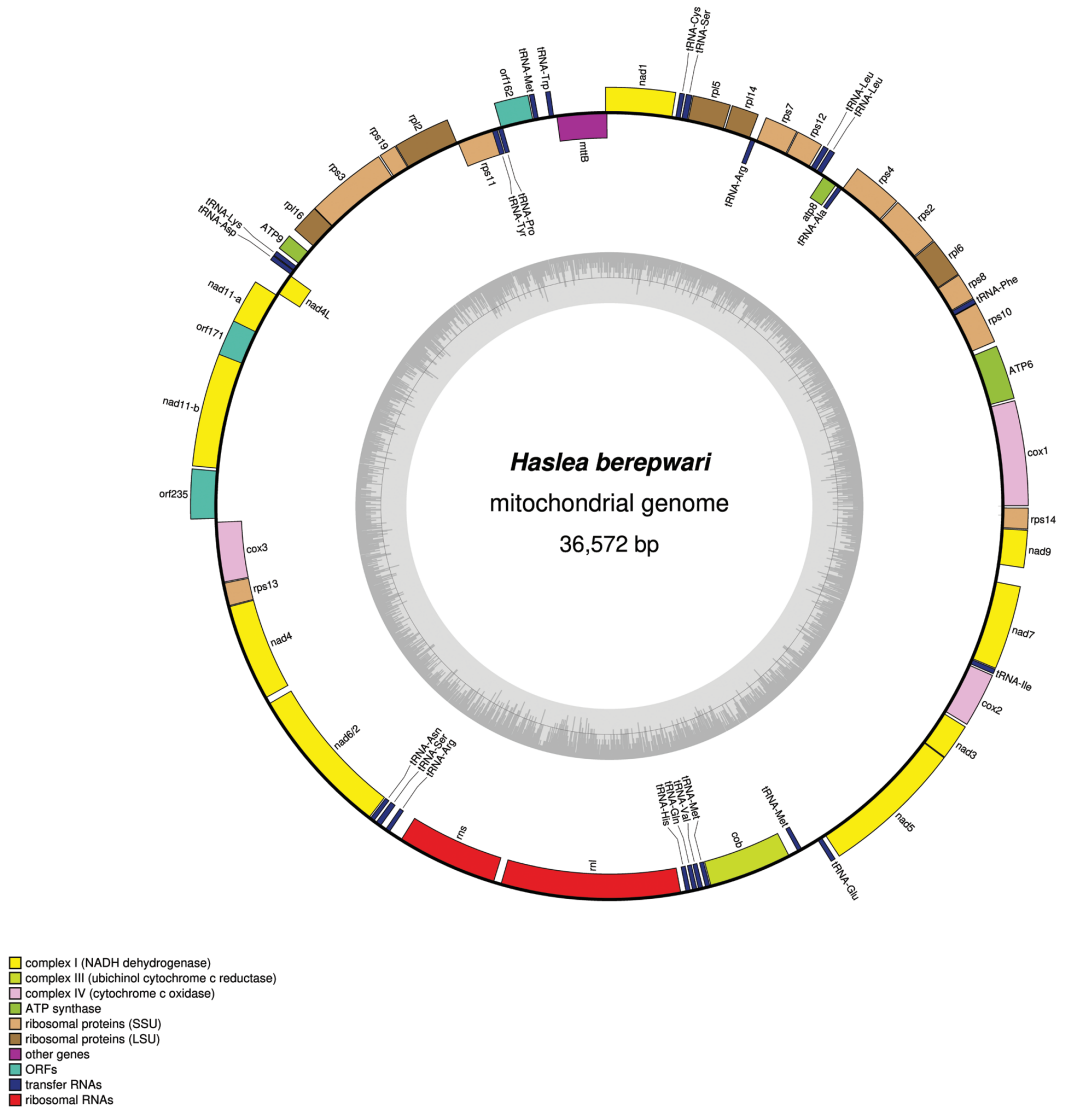
Mitochondrial genome of *Hasleaberepwari* sp. nov. Coloured boxes represent genes, with colours denoting their categories as indicated in the bottom left legend. The inner grey ring shows variations in G+C content.

***Mitochondrial genome***: The mitochondrial genome of *H.berepwari* was retrieved from the contigs file with redundant endings. After trimming and circularization (Fig. 4), its length is 36,572 bp (GenBank: PP728232). The mitogenome encodes for 34 proteins, considering that *nad11* is split into two distinct subunits. As it was noticed with other species of *Haslea* spp., *nad6* and *nad2* are merged into a single open reading frame (ORF), for a total size of 753 amino acids (Gastineau et al. 2021b; Dąbek et al. 2022). The mitogenome also encodes for three ORFs. The first one, *orf162*, corresponds to the conserved ORF generally found *mttB* and *rps11* (Pogoda et al. 2019; Dąbek et al. 2022). We note that our annotation software (Gagnon 2004) ascribed it to *rpl10*, a function suggested for this ORF in *Pleurosigma* sp. (QYJ09263) (Wang et al. 2022). However, in the absence of more evidence of the function of this gene, we will keep labelling it as orf162, nothing also that the size of the putative protein encoded is identical among all the species of *Haslea* spp. for whom a mitogenome is available. The two other ORF, namely orf171 and orf235, are interspersed between the two subunits of *nad11* and *cox3* and are similar to ORFs found in the same position among other species of *Haslea* spp. (Gastineau et al. 2021b; Dąbek et al. 2022). InterProScan queries returned no results for orf235. For orf171, four transmembrane domains, three cytoplasmic regions and two non-cytoplasmic domains were found. The mitogenome also encodes 22 tRNA and two ribosomal rRNA.

**Figure 5.**
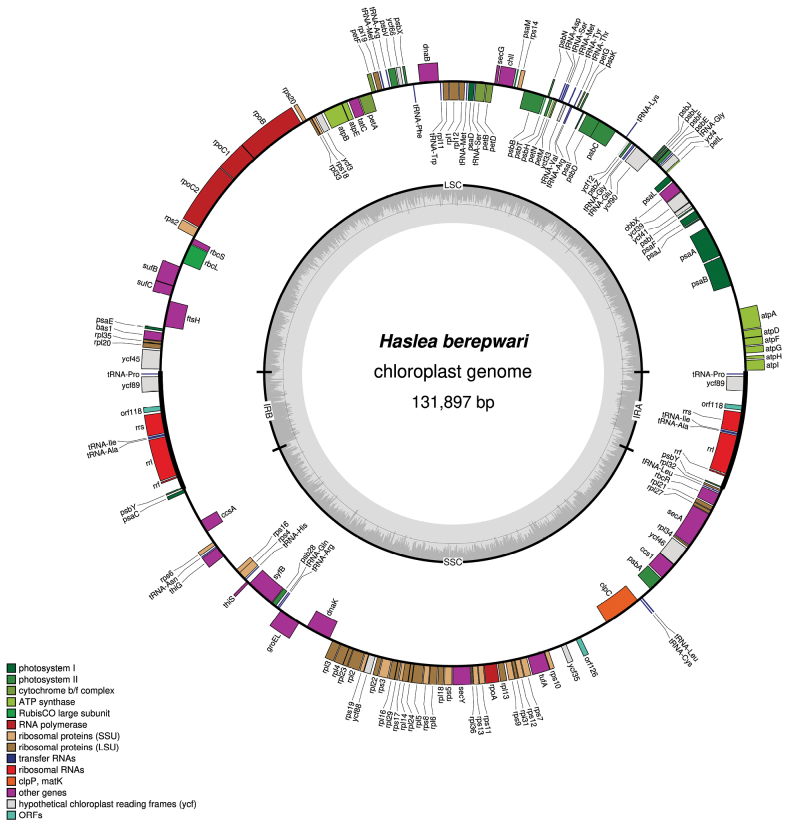
Plastid genome of *Hasleaberepwari* sp. nov. Coloured boxes represent genes, with colours denoting their categories as indicated in the bottom left legend. The inner grey ring shows variations in G+C content.

***Plastid genome***: The plastid genome is 131,897 bp long (GenBank: PP728231) and exhibits the usual quadripartite structure (Fig. 5). The LSC is 65,599 bp long and contains 74 protein-coding genes and 17 tRNA. The SSC is 48,934 bp long and contains 52 protein-coding genes, a single non-conserved ORF and seven tRNA. The inverted repeats are 8,682 bp long and contains two protein-coding genes, a non-conserved ORF (orf118), three rRNA genes and three tRNA. The noticeable differences when compared to *H.pseudostrearia* are the position of *cplC* (between *psbA* and *ycf35*) and the absence of overlap between *ycf45* and the IRB.

**Figure 6.**
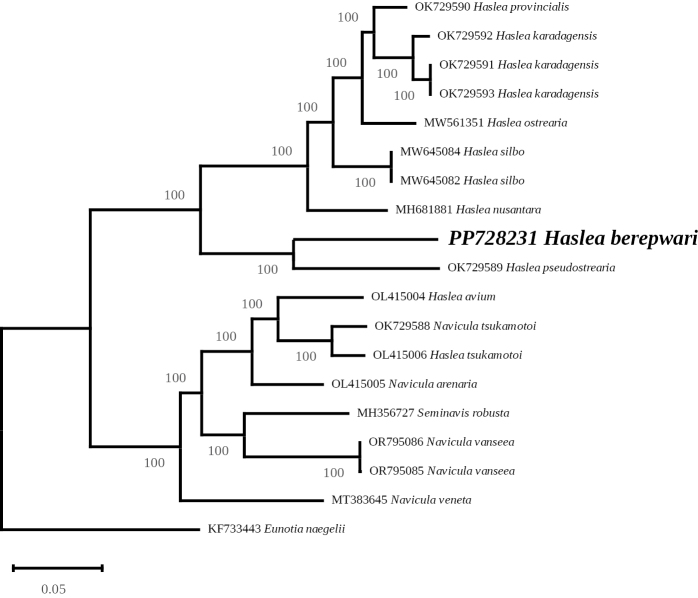
Maximum Likelihood phylogenetic tree obtained from concatenated alignments of 123 protein coding genes from 19 species of diatoms. The tree is rooted with *Eunotianaegelii*.

**Figure 7.**
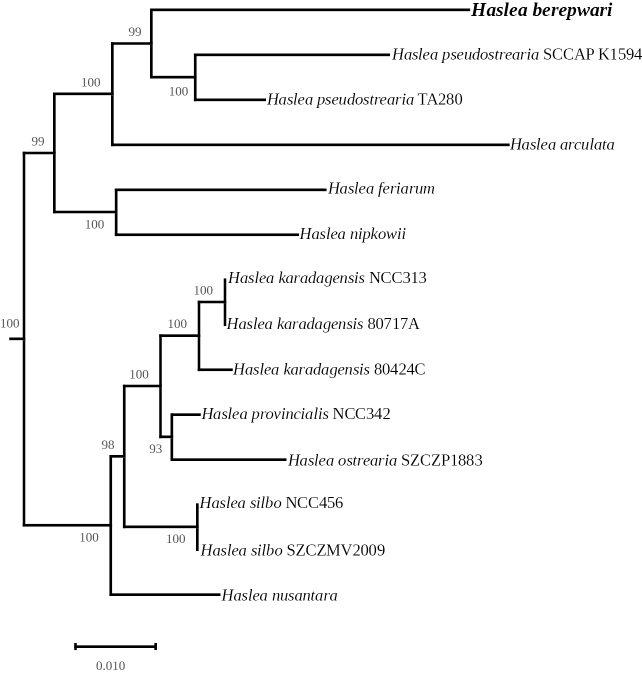
Maximum Likelihood phylogenetic tree obtained from concatenated alignments of three protein coding genes from 133 species of diatoms. The tree was rooted with *Triparmapacifica*. The subtree containing the 14 species of *Haslea* spp. is shown.

***Multigene phylogeny***: The 123-genes ML phylogeny led to a highly supported tree in which all nodes display maximum support (Fig. 6). For the genus *Haslea*, the tree distinguishes between a highly supported clade of marennine-like producing species and a second clade that contains *H.berepwari* sp. nov. and *H.pseudostrearia*. It is noteworthy that the genetic distance between both species is rather important when compared to the distance between ‘blue’ species. Other taxa registered as *Haslea* on GenBank are nested within *Navicula* spp., but their belonging to the genus *Haslea* has been invalidated in Li et al. (2017) and thus should be instead regarded as *Navicula* spp. It is to note that *Seminavisrobusta* D.B.Danielidis & D.G.Mann 2002 appears inside the *Navicula* clade, a position already observed in the 3-genes ML phylogeny recently published in Yılmaz et al. (2024a). The three-genes ML phylogeny (Fig. 7) also associated *H.berepwari* to a clade formed by two strains ascribed to *H.pseudostrearia* with high support. Sister to this clade is *Hasleaarculata* Lobban & Ashworth, 2020, a species found in the Island of Guam and which is characterized by the curved shape of its frustule (Lobban et al. 2020). This large clade is sister to the sigmoid species *Hasleanipkowii* (Meister) M.Poulin & G.Massé 2004 (Poulin et al. 2004) and *Hasleaferiarum* M.A. Tiffany & F.A.S. Sterrenburg 2015, a species with dorsoventral valve shape (Sterrenburg et al. 2015; Li et al. 2017). The tree strictly separates ‘blue’ and ‘non-blue’ taxa.

## ﻿Discussion

### ﻿Comparison between *H.berepwari* and similar species

Morphologically as well as phylogenetically, *H.berepwari* is very similar to *H.pseudostrearia*. We can cite the shape of the external distal raphe ending, which is curved in both taxa. This characteristic is also present among other species such as, for example, *Hasleasalstonica* Massé, Rincé & E.J.Cox 2000 or *Hasleacrucigera* (W.Smith) Simonsen 1974 (Massé et al. 2001). However, it has never been observed among any of the ‘blue’ species so far, and for this reason, it can be regarded at least as a relevant criteria to discriminate between *H.berepwari* and the ‘blue’ species *H.nusantara*, for example. The presence of a central thin bar close to the internal proximal raphe ending is an interesting character too, but that could also be misleading. This thin bar is exhibited by *H.berepwari* and *H.pseudostrearia*, but also by the ‘blue’ species *Hasleasilbo* Gastineau, Hansen & Mouget, 2021 and *H.nusantara*, while it is completely absent in *H.ostrearia*, *H.karadagensis* and *Hasleaprovincialis* Gastineau, Hansen & Mouget, 2016 (Gastineau et al. 2012a, 2016, 2021a; Prasetiya et al. 2019b). So far, the difference in the density of longitudinal striae between *H.berepwari* and *H.pseudostrearia* seems to be the most reliable morphological character to distinguish between both species.

### ﻿Evidences for a ‘*pseudostrearia*-like’ clade among the genus *Haslea*

When they described *H.pseudostrearia*, Massé et al. (2001) noted its similarities to *H.ostrearia*, which influenced their choice of the name. At that time, their conclusions were based solely on morphology, as no molecular analyses were conducted. Later, the genetic proximity between these two species was assessed by the nuclear 18S-inferred phylogeny of Damsté et al. (2004) and, to some extent the plastidic 16S-inferred phylogeny of Poulin et al. (2004). However, two points need to be underlined. First, the sampling of properly identified *Haslea* spp. was of four taxa in Damsté et al. (2004). Poulin et al. (2004) identified six taxa at the species level plus one labelled as ‘*Haslea* sp.’. None of these studies included more than one single blue species, as it predates the description of *Hasleakaradagensis* Davidovich, Gastineau & Mouget in 2012 (Gastineau et al. 2012a). It should be noted that in Poulin et al. (2004), with an increased sample of species, *H.pseudostrearia* clusters with the aforementioned *Haslea* sp. As a molecular marker, 16S has not been widely employed among diatoms, so comparisons were limited until complete plastid genomes had been published. Out of curiosity, the 16S gene of this *Haslea* sp. (AF514851) was submitted to a megablast query. It appears that after itself, the best result returned is *H.berepwari* (1324/1332 bp identical), before *H.pseudostrearia* SCCAP K-1594 (1321/1332 identical) or *H.ostrearia* (1320/1332 bp identical). There is limited information available regarding this *Haslea* sp., except that it originates from the Bay of Bourgneuf, France (Poulin et al. 2004). In the absence of further data on this strain, we can only hypothesize that there may be several species, worldwide and from very distinct environments, that could belong to a ‘*pseudostrearia*-like’ clade, warranting further investigation. The three-genes tree provides additional support to this hypothesis. Indeed, the two strains ascribed to *H.pseudostrearia*, although clustering together, are characterized by an important genetic distance between them, consequently larger than what can be observed between the two strains of *H.silbo* or the three strains of *H.karadagensis* and even more important than the distance separating *H.ostrearia* from *H.provincialis*. We hope that in the future, we will be able to investigate more ‘*pseudostrearia*-like’ taxa, with the same protocol as employed here.

### ﻿Biodiversity of New Caledonian diatoms

The study of freshwater diatoms in New Caledonia can be traced back to the early 20^th^ century (Pearson et al. 1922), at a time where the only available tool would be LM. With the development of SEM, the flora had been reinvestigated by Pr. René Le Cohu (1985), who, along with colleagues, continued to explorer the freshwater taxa (Le Cohu et al. 2018, 2020a, 2020b; Marquie et al. 2018). In contrast, research on marine diatoms has been more recent, relatively limited and was conducted by Dr. Catherine Riaux-Gobin and colleagues. Similar to the current article, their studies have highlighted the biodiversity of this region, sometimes leading to the description of new species and genera (Riaux-Gobin et al. 2022a, 2022b). New Caledonia also possess a coral reef, and such environments are known to host very diverse diatom assemblages (Risjani et al. 2021). For all these reasons, we might advocate for a more systematic investigation of New Caledonian diatom biodiversity. Such investigations should employ a protocol that integrates morphological and molecular (if not genomic) analyses. This approach would enhance our general knowledge on South Pacific diatoms and contribute to the development of accurate and reliable databases for coastal biomonitoring. Additionally, it is worth noting that this diatom was isolated from an aquaculture facility, and such facilities could also benefit from the bioprospection of local diatoms, for example, as feed sources for larval stages of artificially grown organisms

## Supplementary Material

XML Treatment for Haslea berepwari

## ﻿Additional information

### Author ORCIDs

Fiddy Semba Prasetiya https://orcid.org/0000-0001-6474-2319

Thierry Jauffrais https://orcid.org/0000-0001-9681-6239

Mustapha Mohamed-Benkada https://orcid.org/0000-0003-1865-3854

Nolwenn Callac https://orcid.org/0000-0002-7578-3455

Elif Yılmaz https://orcid.org/0000-0001-7939-1814

Claude Lemieux https://orcid.org/0000-0001-9580-8042

Monique Turmel https://orcid.org/0000-0001-7060-035X

Jean-Luc Mouget https://orcid.org/0000-0003-0154-7289

Danang Ambar Prabowo https://orcid.org/0000-0001-8200-1266

Debora Christin Purbani https://orcid.org/0000-0001-9305-2835

Diah Radini Noerdjito https://orcid.org/0000-0002-3274-7307

Varian Fahmi https://orcid.org/0000-0002-4273-9087

Sulastri Arsad https://orcid.org/0000-0002-7322-7834

Romain Gastineau https://orcid.org/0000-0001-8661-5118

### Data availability

All of the data that support the findings of this study are available in the main text. All the sequences derived from next generation sequencing are available on GenBank with accession numbers PP725422, PP728232 and PP728231. Sequencing reads have been deposited on the Sequence Read Archive (SRA) with accession number SRR31857227, BioProject PRJNA1199224 and BioSample SAMN45869835. Original LM and SEM pictures plus the sequences obtained by PCR and Sanger sequencing are available on Zenodo following this link: https://doi.org/10.5281/zenodo.14506379. The dataset for the three-genes phylogeny, the partition file used and the complete tree obtained are all available following this link: https://doi.org/10.5281/zenodo.14931226.
